# Supplementary material for: Brief daily functional strength training to improve functional performance in older adults with mobility disability: A randomized trial
Source: PLoS One. 2026 Mar 12;21(3):e0336748. doi: 10.1371/journal.pone.0336748 (PMC12981469; doi:10.1371/journal.pone.0336748)
Supplement: S2 File — (DOCX) [file pone.0336748.s002.docx]

**Protocol Title: Brief daily functional strength training to Improve functional performance in older adults with mobility disability: A randomized trial**

**Principal Investigator:**

Name: Christopher Sciamanna, MD, MPH

Department: Department of Medicine, Department of Public Health Sciences

Institution: Penn State College of Medicine

Location: Hershey, PA, USA

E-mail Address: cns10@psu.edu

**Clinicaltrials.gov Registration #** NCT05697497

**Background**

Difficulty with walking represents the first stage of decline in physical function, as it is typically the first activity limitation that older adults develop. “Serious difficulty walking or climbing stairs”, referred to as “mobility disability” leads to larger declines in quality of life than depression, anxiety, or pain and to significant increases in financial stress. Older adults with mobility disability are 8.7 times more likely to die, incur an additional $10,000 each year in health care costs and are 13-36 times more likely to transition to a nursing home in the near future.

Resistance training (RT) improves mobility disability but too few older adults do it. Systematic reviews observe that 6 months of RT increases strength by 50% in older adults, which improves mobility (Cohen’s d=0.61, 5 trials). Despite these identified benefits, fewer than 20% of older adults meet national guidelines for doing RT twice per week. There is a clear need to create RT options for older adults that improve physical function and that most are willing to do.

Our team has observed that 84% of older adults with difficulty walking preferred doing RT 5 minutes per day versus the traditional 45 minutes three-times weekly in part due to physical limitations and pain. Briefer RT sessions are also supported by evidence that aerobic exercise high-intensity interval training leads to large increases in fitness in as little as 3 minutes per week, while systematic reviews observe that most of the strength gains are from the first few sets each week. These prior studies suggest that longer programs that include RT may not always be necessary for health benefits and might discourage participation among people with mobility challenges.

Our goal, is to design a brief RT program that can improve physical function among older adults. We have designed a program called FAST (Functional Activity Strength Training) augmented with goal-setting, rarely used in RT studies, for the number of additional repetitions participants should be able to do over time. Our first study in 24 healthy older adults (FAST-1) included prescribing 30 seconds of squats and push-ups each day with given no personal supervision. Over six months, participants performed the exercises on 73% of days and showed large increases in squat performance (+6.2 repetitions, Cohen’s d > 1.0). This study lacked a control group and evaluated only self-reported outcomes. In the current study (FAST-2), we build on this study to test whether FAST can, in 4 minutes of RT per day, improve physical function among older adults with mobility disability

**Study Hypothesis**

We hypothesize that participants randomized to the FAST-2 treatment intervention will improve measures of lower extremity performance that are strongly associated with future disability, in 12 weeks, compared to those randomized to the delayed treatment control.

**Primary Study Endpoints**

| **Measure** | **Timing** |
| --- | --- |
| Five-Times Sit-to-Stand (FTSTS) test | Baseline, week 6 and week 12 |
| One-Legged Stance Test (OLST) | Baseline, week 6 and week 12 |
| 30 second chair test | Baseline, week 6 and week 12 |
| Exercise daily performance | Daily for 12 weeks |

## Secondary Study Endpoints

| **Measure** | **Timing** |
| --- | --- |
| Adherence reporting | Daily |

#

**Ethics Approval and Informed Consent**

Study protocols were reviewed and approved by the Penn State University Institutional Review Board (IRB # STUDY00016054). Written informed consent will be obtained from all participants. All research will be conducted in accordance with the Declaration of Helsinki, Good Clinical Practice guidelines and Penn State Health local regulatory requirements. The study will followthe Consolidated Standards of Reporting Trials (CONSORT) reporting guideline and has been registered at [www.clinicaltrials.gov](https://www.clinicaltrials.gov/) under the identification number NCT05697497.

**Methods**

**Participants**

Eligible participants will be identified through mailing recruitment letters to Penn State Health patients ≥ 65 years of age meeting eligibility criteria and residing in zip codes within a 30-mile radius of Penn State Health-Milton S. Hershey Medical Center.

Eligibility screening process

Screening for eligibility will occur prior to the signing of the informed consent document. Interested participants will be screened by phone by research staff who will read a phone screening document which will ensure eligibility and inform them of study procedures.

During screening, the items from the Physical Activity Readiness Questionnaire (PAR-Q; attached) will be asked. The participant answers will be transferred to the permission slip (attached) by research staff and will be faxed to the Primary Care Provider (PCP) of the participant for the PCP to review and provide permission.

**Inclusion and Exclusion Criteria**

**Inclusion Criteria**

1. Age 65 and older
2. Difficulty with walking
3. Providers must provide consent for their patient
4. Access to the internet
5. Must have a camera on computer, tablet, or smartphone
6. Fluent in English

**Exclusion Criteria**

1. Chest pain or a heart condition on the PAR-Q
2. Planning to have surgery in the next 3 months
3. Cognitive impairment
4. Unable to exercise
5. Being currently physically active

## Study Procedures

## Randomization and blinding

Participants will be randomized through REDCap using stratified assignment based on biological sex and age (65-72 and 73+) to maintain equal representation of older vs. younger males and females in each group. Once randomized, patients will be informed of their assigned condition, and their study visits will be scheduled. Both participants and the research staff who conduct the assessments will not be blinded to the participants’ treatment assignments. Secondary coders that review videos weekly to assess quality and for safety control will be blinded.

**Intervention**

FAST-2 will be a two-group, 12-week, delayed-treatment randomized trial.

Participants will be provided with a set of four resistance bands with handles (10-40 pounds of resistance) and a standard aerobics stepper that can be adjusted to 4, 6, or 8 inches in height prior to the baseline visit. They will be instructed to perform four exercises daily, each lasting 30 seconds, and to perform as many repetitions as possible during those 30 seconds. Thirty seconds of rest will be allowed in between the exercises. Each day participants will be asked to perform push-ups, chair stands, two-arm rows, and stair stepping in the same order during each session. No written instructions will be provided, although links to videos of each exercise will be included in email communications. Participants will be instructed to perform the exercises all seven days of the week. Modifications will be provided based on the participants’ functional level and form.

Push-ups can be modified in any of the following ways: by resting on the knees instead of the toes, by placing the hands on the kitchen countertop (30 inches in most homes), by placing the hands on a set of steps (starting with the 4^th^ step from the floor) or by placing the hands on the wall. When participants are able to perform 15 repetitions of push-ups using one of the modified methods, they will beasked to progress to a higher level of difficulty (e.g., place the hands on the 3^rd^ step from the floor).

Chair stands will be performed, by default, by placing the arms across the chest, but participants can modify them if needed by placing two hands on the knees, one hand on a knee or with arms not placed across the chest. Participants will be instructed to use a standard chair with arms, without wheels and with a seat height of approximately 17 inches regardless of the height of the subject. Participants will be encouraged to progress, as they are able to do so safely, to the default position of placing their arms across the chest.

Two-arm seated band rows will be performed with the resistance band looped around the arches of the feet to allow for a full range of motion. Participants will be instructed to pull their elbows back to touch the body to complete a repetition while squeezing the shoulder blades behind together.

Participants will complete the stair stepping exercise by placing their feet up on the step reciprocally and then stepping backwards down in the same fashion. All participants will start on the 4-inch step until they can rise fully on the step with both feet six times in 30 seconds. They will be then instructed to increase the step height to 6 inches and later to 8 inches.

To increase safety, participants will be instructed to place the chair on carpet or against a sturdy object like a wall. For the stair stepping exercise, participants will be instructed to place the stair stepper in the corner of a room, so they can touch the wall if they feel unsteady.

### Coaching

At baseline and at weeks 2, 4 and 8, each participant will complete a one-on-one Zoom audio-video coaching session led by a research staff member with expertise in exercise and health psychology with the goal of improving each participant’s personal performance records. Each coaching session will last 10-20 minutes, depending on the participants’ instructional needs. The coach will begin each session by asking the participant a question designed to build rapport (i.e., “small talk”). Next, the coach will review the self-reported adherence and performance data of participants, congratulating them on any personal records and positive performance trends. The coach will emphasize to the participants the importance of moving as rapidly as possible (without sacrificing form) to pursue a standardized goal of increasing chair stands and stair steps, by four and two repetitions, respectively over the course of the 12-week intervention. The same goal will be communicated by the coach to all participants. The stated rationale of this goal is to improve walking ability, as all participants have reported difficulty walking at baseline. Before beginning the exercises, the participants will orient their camera so that the entire body will be visible by the coach. During each session, the coach will passively observe the participant complete the entire workout. The coach will ask each participant to complete the workout normally, as if they are not being observed. Upon completing the 4-minute exercise session, the coaches will provide feedback and correction (if necessary) relating to exercise form. Where form correction is necessary, the coach will demonstrate the proper movement and confirm understanding by viewing the participant perform the corrected exercise. Modifications and progression methods will be suggested and discussed. For example, once a participant can complete 15 push-ups with their hands on a kitchen countertop, the coach will encourage the participant to progress to push-ups with their hands on the 4^th^ step of a staircase (if available in the home of the participant). Safety will be emphasized during each session; coaches will encourage participants to place the stepper in a corner (to allow them to regain their balance, if needed) and to do chair stands on a carpeted floor, so the chairs will not slide. The coach will end each session by answering any questions.

### Self-monitoring, feedback, and messaging

Each participant will receive an email reminder every morning to complete the daily workout along with a link to a REDCap survey to report their performance on the four exercises (number of push-ups, chair stands, rows and steps on the stepper), whether they used exercise modifications, and their perception of exertion during the workout. Emails will include links to videos of each exercise. Every other week, participants will receive a separate email summarizing their performance and adherence and recognizing their new personal best performance.

## Delayed intervention control

Participants assigned to the delayed intervention control group will continue care as usual. Following the completion of the 12-week follow-up, participants in the delayed group will begin 12 weeks of physical activity intervention with a one-on-one audio-video Zoom coaching session occurring during the initial first week. At that time, the information given to the physical activity treatment intervention group regarding the daily exercise will be given to the delayed group.

**Measures**

**Primary Outcome Measures**

**Functional Performance Measures**

The participants in both groups will complete three functional performance measures while being observed by a trained researcher over Zoom at baseline, week 6 and week 12 (The Five-Times Sit-to-Stand (FTSTS) test, part of the Short Performance Physical Battery(SPPB)), the One-Legged Stance Test (OLST) and the 30 second chair stand test. As with the coaching sessions, the participants will orient their camera so that the entire body will be visible to the researcher

**Five-Times Sit-to-Stand (FTSTS) test**

Participants will be instructed to sit in and fully rise from a chair five times as quickly as possible, without using their arms for support. The test which will be timed using a stopwatch will end when the participant’s body touches the chair following the fifth repetition.

**One-Legged Stance Test (OLST)**

Subjects will be instructed to start with a comfortable base of support, with both eyes open and arms by their side and then stand unassisted on the right leg. The OLST will be measured from the time that the left foot is lifted from the floor to when it touches the ground or the other leg).

**30 second chair stand test**

Participants will be instructed to sit in and fully rise from a chair as many times as they can in 30 seconds.

The OLST will be performed after the FTSTS and before the 30 second chair stand test, to reduce lower extremity fatigue.

**Daily exercise performance**

Participants will enter their push-up, chair stand, row and step-up exercise repetitions, whether they used exercise modifications, and how difficult they felt the workout was using a survey sent daily through REDCap.

Videos will be reviewed weekly by a blinded secondary coder to measure quality and safety control for the exercises performed. All timed outcome measure performances will be immediately recorded during the Zoom session at the conclusion of each functional performance measure and re-evaluated offline by a blinded reviewer. Discrepancies in recorded times that are greater than one second will be replaced with the time recorded by the secondary reviewer. If discrepancies in recorded times are less than 1 second, the average of the two values will be used. If there is a disagreement on the number of repetitions performed between the blinded secondary coder and the unblinded research staff member conducting the exercise session, the blinded staff member will provide the final adjudication.

### Secondary Outcome Measure

### Adherence and retention

An exercise session will be determined to have been completed only if the participants complete the daily REDCap survey reporting their performance, exercise modification use and difficulty level.

If participants do not complete their daily exercise sessions for ≥ 3 days, research staff will attempt to contact the lapsed participants via phone and email up to 5 times to re-engage them. Participants will be contacted every 48 hours until 14 days of missed daily participation. Participants who do not respond to the project staff for ≥ 14 days will be treated as unable-to-be-contacted with no further contact attempts made unless the participant re-establishes contact on their own.

## Sample size determination

Effect size calculations are based on evidence that 30 second chair stand performance declines with age and predicts future functional decline. We considered a large effect size as healthier patients in our pilot showed large gains (i.e., *d > 1.00)* in squat performance, but because our intended sample has at least some degree of limitations with walking, we expect a more conservative effect of the intervention on measures of physical function (i.e., d = .60).

Based on a 2-group design (intervention, control) assuming a moderate standardized between-group difference at 12 weeks (Cohen’s d = .60), a sample size of 88 (44 per group) would be needed to detect a difference between groups with a two-sided significance level of 0.05 and 80% power. To account for an anticipated 20% attrition rate over the study period, we will recruit up to 110 participants.

## Statistical analysis

Summary statistics (e.g., mean and standard deviation (SD) for continuous variables; frequency and proportion for categorical variables) will be reported for participant baseline characteristics. Baseline differences between randomly assigned participants will be evaluated with two-sample t-tests and Fisher’s exact tests. Changes in the primary outcome measures over 12 weeks will be evaluated at the group level using linear mixed-effect models to analyze change over time and the Group x Time interaction. The analysis will employ an intention-to-treat approach, with missing data addressed through mixed-effects modeling in the longitudinal data analysis framework. A two-sided *p*-value of less than or equal to .05 will be considered statistically significant.

### Criteria for removal from study

Participants may be from the study if they become injured and cannot exercise or if they are asked by their Primary Care Provider to suspend exercise. The Principal Investigator reserves the right to remove a participant from the study for any reason, based on their discretion, particularly a belief that continued participation exposes them to unnecessary risk. Also, participants have the right to withdraw themselves at any time and halt all study-related activities permanently.

Other withdrawal criteria include: unwillingness to follow protocol during visits, unwillingness to complete study visits, and/or return of equipment to study staff, withdrawal of consent by the participant, development of a new medical problem that would exclude them from the study.
