# Supplementary material for: Brief daily functional strength training to improve functional performance in older adults with mobility disability: A randomized trial
Source: PLoS One. 2026 Mar 12;21(3):e0336748. doi: 10.1371/journal.pone.0336748 (PMC12981469; doi:10.1371/journal.pone.0336748)
Supplement: Attachment — Submitted filename: Response to Reviewers.pdf [file pone.0336748.s004.pdf]

December 19th, 2025,

Dear Editor,

We want to thank you and the reviewers for taking the time to review our manuscript titled "Brief daily functional strength training to improve functional performance in older adults with mobility disability: A randomized trial" where we provide experimental evidence that a 12-week (4-minute) , home-based functional resistance training program augmented with goal setting, FAST (*Functional Activity Strength Training)*-2, can enhance adherence and improve functional impairment in older, inactive adults ≥ 65 years of age, with pre-existing walking difficulty. We greatly appreciate your thoughtful and constructive comments, which provide valuable insights to strengthen the submission. We have carefully considered each point and incorporated revisions accordingly which are mentioned in red in the body of the manuscript.

Please find our detailed responses to your and the reviewers' comments below.

1.Please ensure that your manuscript meets PLOS ONE's style requirements, including those for file naming. The PLOS ONE style templates can be found at

https://journals.plos.org/plosone/s/file?id=wjVg/PLOSOne_formatting_sample_main_body.pdf and

https://journals.plos.org/plosone/s/file?id=ba62/PLOSOne_formatting_sample_title_authors_affiliations.pdf

Response: *We have reviewed the entire manuscript and ensured that it meets PLOS ONE's style requirements. A change was made to the affiliation of the first author (Line 14) and the font of a level 2 heading (Line 129)*

2. We note that you have selected "Clinical Trial" as your article type. PLOS ONE requires that all clinical trials are registered in an appropriate registry (the WHO list of approved registries is at https://www.who.int/clinical-trials-registry-platform/network/primary-registries" https://www.who.int/clinical-trials-registry-platform/network/primary-registries and more information on trial registration is at http://www.icmje.org/about-icmje/faqs/clinical-trials-registration/). Please state the name of the registry and the registration number (e.g. ISRCTN or ClinicalTrials.gov) in the submission data and on the title page of your manuscript. a) Please provide the complete date range for participant recruitment and follow-up in the methods section of your manuscript. b) If you have not yet registered your trial in an appropriate registry, we now require you to do so and will need confirmation of the trial registry number before we can pass your paper to the next stage of review. Please include in the Methods section of your paper your reasons for not registering this study before enrolment of participants started. Please confirm that all related trials are registered by stating: "The authors confirm that all ongoing and related trials for this drug/intervention are registered". Please see http://journals.plos.org/plosone/s/submission-guidelines#loc-clinical-trials for our policies on clinical trials.

Response: *We have added the registry name (ClinicalTrials.gov) and the trial registration number on the title page of the manuscript (Lines 37-38). We previously referenced it in the submission data which remains in the manuscript (Lines 507-509). Additionally, the name of the registry and the trial registration number can be found in the Materials and methods section (Lines 137-139). The complete date range for participant recruitment and follow-up was included in the methods section of the manuscript and remains the same (Lines 157-159)*

3. In the online submission form, you indicated that “The data that support the findings of this study are available upon request to the correspondent author”

All PLOS journals now require all data underlying the findings described in their manuscript to be freely available to other researchers, either 1. In a public repository, 2. Within the manuscript itself, or 3. Uploaded as supplementary information.

This policy applies to all data except where public deposition would breach compliance with the protocol approved by your research ethics board. If your data cannot be made publicly available for ethical or legal reasons (e.g., public availability would compromise patient privacy), please explain your reasons on resubmission and your exemption request will be escalated for approval.

Response: *Thank you for the comment. We have changed our data sharing statement in the manuscript and clarified that the data associated with this study will be made available via Penn State Data Commons (Lines 502-504)*

4. Please include your full ethics statement in the ‘Methods’ section of your manuscript file. In your statement, please include the full name of the IRB or ethics committee who approved or waived your study, as well as whether or not you obtained informed written or verbal consent. If consent was waived for your study, please include this information in your statement as well.

Response: *The “Study design” sub-heading under the “Materials and methods” section of the manuscript includes the full ethics statement, including the full name of the IRB who approved the study and the documentation of the receipt of informed written consent as follows “Study protocols were reviewed and approved by the Penn State University Institutional Review Board (IRB # STUDY00016054). Written informed consent was obtained from all participants. All research was conducted in accordance with the Declaration of Helsinki, Good Clinical Practice guidelines and Penn State Health local regulatory requirements” (Lines 133-136).*

5. Thank you for stating the following in the Competing Interests section:

“The author(s) declared the following potential conflicts of interest with respect to the research, authorship, and/or publication of this article: Dr. Sciamanna is part-owner of BandUp, Inc. and Play Fitness, LLC, formed to test the viability of various business models to disseminate findings from exercise studies. Dr. Jonathan Stine receives or has received research support from Astra Zeneca, Galectin, Kowa, Noom, Inc, Novo Nordisk, Regeneron and Zydus Therapeutics. Dr. Stine consults for Novo Nordisk and is on an advisory board for Madrigal. No other authors have competing interests.”

Please confirm that this does not alter your adherence to all PLOS ONE policies on sharing data and materials, by including the following statement: "This does not alter our adherence to PLOS ONE policies on sharing data and materials.” (as detailed online in our guide for authors http://journals.plos.org/plosone/s/competing-interests). If there are restrictions on sharing of data and/or materials, please state these. Please note that we cannot proceed with consideration of your article until this information has been declared.

Please include your updated Competing Interests statement in your cover letter; we will change the online submission form on your behalf.

Response: *We have updated the Competing Interests statement to include the statement "This does not alter our adherence to PLOS ONE policies on sharing data and materials.” (Lines 500-501). We have also updated the Competing Interests statement in our cover letter.*

6. If the reviewer comments include a recommendation to cite specific previously published works, please review and evaluate these publications to determine whether they are relevant and should be cited. There is no requirement to cite these works unless the editor has indicated otherwise.

Response: *We have cited our previously published work in the manuscript. There were no requests made by any of the reviewers for additional citations.*

Additional Editor Comments:
(PLACE INSERTS IN A DIFFERENT COLOR FONT TO IDENTIFY CHANGES IN THE ARTICLE)
PUT IN RED LETTERS - ANSWER THE QUESTIONS BELOW POINT BY POINT.
INCLUDE THE PAGE AND LINE WHERE YOU ARE MAKING THE CHANGE.
PLEASE INCLUDE ALL REQUESTS IN THE MANUSCRIPT.
Include in article
0 - Please correct grammatical and spelling errors
1 - Abstract
Conclusions: State only what your study found; do not include extraneous information not backed up by the results.

Response**:** *We have made the change as suggested and omitted part of the last sentence which suggested clinical benefit but was not backed by the results (Line 82)*

2 - Discussion
Compare and contrast your study with others in the most relevant world literature, particularly the recent literature.

Response: *Thank you for this comment. We have compared and contrasted our study with the most relevant and recent literature. This is included in the Discussion section over multiple paragraphs (Lines 387-430)*

3 - What new information is sufficient to modify existing clinical practice?

Response:
We have included in the discussion new information resulting from our research that offers evidence that a brief, home based, functional RT program could prevent and improve functional limitations in older adults, potentially changing the trajectory of their mobility disability. The results of our study sufficiently support modifying existing clinical practice and clarify that brief RT programs could remove barriers to disseminating RT and improve older adults' physical function.

"In this randomized trial of older adults with pre-existing walking difficulty, 12 weeks of a 4-minute brief, home based, daily, functional RT program called FAST (Functional Activity Strength Training)-2, which included only 60 seconds of lower extremity RT exercise yielded significant improvement in functional performance compared to usual care. Moderate to large differences between groups were observed, with the RT group improving by 4.2 more repetitions than the Control group in the 30 second chair stand performance (95% CI: 2.8-5.7, $p < 0.001$), 3.6 more seconds more than the Control group in their OLST time (95% CI: 0.6-6.5, $p = 0.02$) and a decrease of 2.3 seconds relative to the Control group on the FTSTS test (95% CI: 0.5-4.1, $p = 0.01$). This is clinically significant as the reported Minimum Clinically Important Difference (MCID) for the 30 second chair stand test is ≥ 2 repetitions for predicting improvement in the 6-minute walk test(38) and the MCID for FTSTS is 2.3 seconds.(39) These results are also consistent with findings in RT literature in general, which note that the first few sets of exercise per week lead to 80% of the gains in strength as higher set volumes.(23) Furthermore, the FAST-2 program had a high level of adherence and uptake, with those assigned to the intervention completing the exercises on an average of 81% of days (5.6 days per week). Collectively, these results suggest that brief, home based, functional RT programs could prevent and improve functional limitations in this growing population potentially changing the trajectory of their mobility disability." (Lines 387-403)

"Our findings have clinical relevance as it's often challenging to find an exercise program that can be easily adopted and well completed in a population that is often hesitant to begin exercise programs even when offered at no extra cost. Difficulty with walking represents the first stage of decline in physical function in older adults. Our study showed a high level of adherence to the physical activity treatment intervention (81%) over 12 weeks. In a systematic review of home-based exercise programs for older adults with mobility difficulty with an average intervention period of 8-12 weeks, the average rate of adherence was found to be only 67% (12 studies).(40, 41) Apart from a social contract and accountability from the periodic video supervision, no further incentives were provided for study completion. Despite this, adherence remained high, supporting the feasibility of the intervention." (Lines 404-413)

4 -What are the conclusions and implications for current practice, and particularly for future research that may have a significant impact on clinical decisions?

Response: *As stated in the manuscript, our conclusion from our research is that a brief 4-minute daily functional RT program, which included only 60-seconds of lower extremity exercises, showed significant improvement in measures of lower extremity performance that are strongly associated with future disability in older adults. (Lines 467-469)*
In our opinion, future studies are needed to understand whether these differences are maintained with gold-standard measures and whether adherence is maintained over a longer period. If these results are maintained with gold-standard measures and with longer follow-up, this could significantly impact recommendations for strength training and more importantly mobility disability in the elderly population. (Lines 469-471)

5 - How can this study affect public policies related to health?

Response: *The results from our study show that a brief 4-minute daily functional RT program, which included only 60-seconds of lower extremity exercises, can significantly improve measures of lower extremity performance that are strongly associated with future disability in older adults.*
If these results are maintained with gold-standard measures and with longer follow-up, this could significantly impact public policy and recommendations for strength training in the elderly population

6 - What does this study add to the literature?

Response: *As stated in the manuscript,* ***"****Our findings have clinical relevance as it's often challenging to find an exercise program that can be easily adopted and well completed in a population that is often hesitant to begin exercise programs even when offered at no extra cost. Difficulty with walking represents the first stage of decline in physical function in older adults. Our study showed a high level of adherence to the physical activity treatment intervention (81%) over 12 weeks. In a systematic review of home-based exercise programs for older adults with mobility difficulty with an average intervention period of 8-12 weeks, the average rate of adherence was found to be only 67% (12 studies).(40, 41) Apart from a social contract and accountability from the periodic video supervision, no further incentives were provided for study completion. Despite this, adherence remained high, supporting the feasibility of the intervention." (Lines 404-413)*

"The study results extend the existing literature by demonstrating results similar to other studies utilizing brief RT programs, although, with a novel, completely remotely delivered intervention and with minimal supervision. In an uncontrolled study Fujita and colleagues, who exposed older adults to just over 2 minutes of chair stands 3 days per week for 3 months, observed an increase of 18% in knee extension torque.(42) Similarly, in a quasi-experimental study of nursing home residents, Slaughter and colleagues observed that older adults who performed 1 set of repeated chair stands on an average of two times per day experienced a significantly slower decline in sit-to-stand performance and significantly better scores

in the Functional Independence Measure(43) after 6 months.(44) Importantly, these studies were in-person, one-on-one supervised interventions, while the present study was conducted entirely virtually with approximately 30 minutes per month of remotely delivered synchronous video supervision." (Lines 414-425)

7 – Improve - At the end of the Discussion, under the subheading "Limitations," review the limitations of your study.

Response: *Thank you for this comment. We clearly state and include the Limitations of our study in the manuscript.*

"While the study had many strengths, including a randomized design, a population at high risk for future loss of mobility, a cost-efficient and easily disseminable entirely virtual format, high uptake to adherence and a quality control protocol for the three study outcomes, the study had several limitations. First, COVID restrictions curtailed in-person study visits, so the three key outcome measures needed to be collected via video and other gold-standard measures of lower extremity performance (e.g., SPPB, 1 Repetition Maximum, 6 Minute Walk Distance) were not completed. Of note, the SPPB includes the FTSTS, which our study measured, and several balance measures (e.g., tandem stance), while our study included only the OLST. As moderate-large improvements were observed in both the FTSTS and OLST measures, it is quite likely that improvements in SPPB would also have been observed, yet future studies will need to be performed to ensure that FAST improves these gold standard measures. Second, the intervention was only 3 months in duration. While adherence was excellent (81% of days completed), and adherence over 6 months was good (73% of days) in the first FAST study(26), it is possible that older adults will become bored with the same activities over time and, as a result, adherence will decline. Longer studies will be needed to understand whether adherence, as well as performance improvements, persists. Third, as older adults without access to the Internet were excluded, the participants may not be representative of the population at risk. In 2021, 75% of adults over 65 had access to the Internet, but it is unclear what level of access is observed for even higher ages, as the average age in this study was 74.3. Fortunately, 96% of US adults ≥ 50 use the Internet, so lower rates of Internet access today among older adults are likely only temporary.(45) In the near future, nearly all older adults will likely have Internet access. Fourth, the intention-to-treat principle preserves the benefits of randomization, but ITT estimates may underestimate the true effect of the intervention in the presence of nonadherence. Missing outcome data and loss to follow-up, while addressed using linear mixed-effects models under a missing-at-random assumption, may still bias estimates if missingness depends on unobserved factors. Consequently, ITT results should be interpreted as conservative estimates of intervention effectiveness under real-world implementation rather than as estimates of efficacy under full adherence. Fifth, the sample size for the study was small relative to the prevalence of functional limitations in the elderly population. Larger studies will be needed to clarify the external validity of study findings. Finally, it is important to state clearly what this study does not suggest. It does not suggest that four minutes of daily exercise is sufficient to result in body composition or cardiovascular health improvements for the general population; instead, it only suggests that four minutes of daily exercise was sufficient to improve functional performance in older adults with existing walking difficulties." (Lines 432-465)

8 - At the end of the limitations, under the subheading " Future directions".

Response: *We have included the Future directions of our research under the heading "Conclusions". "Future studies are needed to understand whether these differences are maintained with gold-standard measures and whether adherence is maintained over a longer period." (Lines 469-471)*

9 - Conclusion

Take special care to draw your conclusions only from your results and verify that your conclusions are firmly supported by your data.

Response: *Our conclusions are drawn from our results alone. We clearly state in the manuscript, "In conclusion, a brief 4-minute daily functional RT program, which included only 60-seconds of lower extremity exercises, showed significant improvement in measures of lower extremity performance that are strongly associated with future disability in older adults." This is strongly supported by our results which we clearly outline in the "Results" section. (Lines 467-469)*

Reviewer #1: I write to submit my review of the manuscript titled "Brief daily functional strength training to improve functional performance in older adults with mobility disability: A randomized trial."
The study via randomized trial evaluated the effects of a 12-week brief, home-based functional RT program, FAST (Functional Activity Strength Training)-2, on adherence and functional impairment in older, inactive adults ≥ 65 years of age, with pre-existing walking difficulty.

1. Kindly explain what informed age stratifiers of 65-72 and 73+ are? Include justification in the manuscript. Is 65 years the official definition of old age in the USA? Why did you consider only those who were greater than 65 years? Why not 60 years, etc? Kindly include justification for the age inclusion threshold.

Response: *Thank you for the comment. We have clarified in the manuscript our justification for the age including threshold of 65 years and older. Federal programs like Medicare as well as for the National Institute for Aging (NIA) and Centers for Disease Control and Prevention (CDC) define "older adults" as people aged 65 or older which informed our decision to include participants 65 years and older. (Lines 143-146)*

Functional walking limitations rise sharply with age (2). We typically observe that the median age of participants who enroll in our trials is 72-73. For that reason, we selected this as our cut-point on which to stratify randomization. We have clarified our reasoning in the manuscript. (Lines 164-166)

2. What was the rationale behind the 12-week, delayed-treatment randomized trial? What would have been the effect if the duration had been extended beyond 12 weeks or reduced? The rationale for selecting 12 weeks for implementing the intervention should be included in the manuscript.

Response: Thank you for this question. 12 weeks is the typical length of exercise trials. Data suggests that 12 weeks is long enough to allow for physiological changes to occur -- both the neural changes and muscular changes associated with strength training. Initial trials (i.e., those testing a new intervention) typically last 12 weeks to balance participant burden with the ability to test the efficacy of the program. Then, longer trials can be conducted if the 12-week intervention suggested that further investigation was warranted. This has been added to the study design section: "This duration was selected as 12-weeks is a sufficient amount of time to allow for physiological changes to occur." *(Lines 131-133).* Additionally, a reference has been provided (27)

3. Major concern: There was no power analysis (sample size calculation) to determine whether the study was powered enough to assess the effectiveness of the intervention if it exists. No properly estimated sample size. The authors initially screened 415 participants and ultimately arrived at 102 for the main study; however, there is no scientific justification for the final sample size of 102. Would the result have been different if only 60 participants had agreed to participate in the study? More rigorous power analysis needs to be conducted to justify the 120 participants

Response: *Thank you for the comment. A subsection of Sample size determination has been added in the Materials and methods section. The sample size determination previously included in the Trial protocol has been modified to include clarifications. The updated trial protocol is submitted along with the revised manuscript. (Lines 293-303)*

4. For each of the outcome measures, kindly be clear on the measurement scale (continuous, binary, discrete, nominal, ordinal, multinomial outcome) etc. This will determine the appropriateness of the statistical methods employed.

Response: *The three outcome measures: Five-Times Sit-to-Stand (FTSTS) test and One-Legged Stance Test (OLST), and 30 second chair stand test, were all treated as continuous data. Their definitions are given below:*

Five-Times Sit-to-Stand (FTSTS) test

Participants will be instructed to sit in and fully rise from a chair five times as quickly as possible, without using their arms for support. The test which will be timed using a stopwatch will end when the participant's body touches the chair following the fifth repetition.

One-Legged Stance Test (OLST)

Subjects will be instructed to start with a comfortable base of support, with both eyes open and arms by their side and then stand unassisted on the right leg. The OLST will be measured from the time that the left foot is lifted from the floor to when it touches the ground or the other leg).

30 second chair stand test

Participants will be instructed to sit in and fully rise from a chair as many times as they can in 30 seconds.

5. The authors stated that "Summary statistics were reported for participant baseline characteristics". This is too broad. Kindly state the key summary statistics that were reported

Response: *We have elaborated the writing:* "*Summary statistics (e.g., mean and standard deviation (SD) for continuous variables; frequency and proportion for categorical variables) were reported for participant baseline characteristics*." (Lines 305-306)

6. Include key assumptions of the linear mixed effect model and how these assumptions were tested.

Response: *The linear mixed-effects model assumes linearity, normally distributed residuals and random effects with constant residual variance. These assumptions were evaluated using residual-versus-fitted plots and Q-Q plots of residuals and random-effects estimates, with the adequacy of the random-effects structure examined by comparing nested models using likelihood ratio tests. No meaningful violations were observed*. (Lines 311-316)

7. Include the limitations of the intention-to-treat estimates

Response: *We have added the limitations of the intention-to-treat estimates in the last paragraph of the Discussion. "the intention-to-treat principle preserves the benefits of randomization, but ITT estimates may underestimate the true effect of the intervention in the presence of nonadherence. Missing outcome data and loss to follow-up, while addressed using linear mixed-effects models under a missing-at-random assumption, may still bias estimates if missingness depends on unobserved factors. Consequently, ITT results should be interpreted as conservative estimates of intervention effectiveness under real-world implementation rather than as estimates of efficacy under full adherence. Fifth," (Lines 453-459)*

8. Include the mean difference between the intervention and control by creating an additional column before the p-value estimates in Table 1

Response: *We have included an additional column before the p-value estimates in Table 1 as suggested.*

<u>Reviewer #2:</u> The rationale for study is valid and provides an alternative to current recommendations that are needed for older adults, particularly those with a mobile disability. This FAST-2 program is brief, has appropriate progression, and is easily delivered, with internet access. There was strong adherence to the training program and outcomes show improvements in the training group that will improve the ability to complete ADLs and reduce injury in this population. Methodology is sound and the verification of measured outcomes with a second interpreter is a good added control. A few things should be addressed.

1. I am concerned about the two-arm seated band rows and the potential for the band to roll up the foot and injure. Did this happen at all in your study? Can you include an alternate exercise targeting similar muscle groups in the discussion as an alternative in case one is not comfortable doing this?

Response: *We acknowledge your concern regarding the two-arm seated band rows and the potential for the band to roll up the foot and injure. None of our study participants reported any injuries with this exercise. To avoid this possibility, participants were instructed to loop the bands AROUND their feet and keep their feet flat on the floor, thereby tightening the band when they performed the exercise. We have found this method to be very effective at preventing the band from rolling out from under the foot.*

2. The self-monitoring, feedback, and messaging seems potentially laborious for an older person. Was any data collected on how this what utilized by the subjects or how these affected adherence?

Response: The self-monitoring form also included an audio file (with timer) to instruct participants through the exercises. In that way, it was less of an "extra" thing to do, and more of an integral part of the intervention. Participants *reported their performance on four exercises (number of push-ups, chair stands, rows and steps on the stepper), whether they used exercise modifications, and their perception of exertion during the workout using a REDCap survey. The survey was brief lasting less than 5 minutes each day.*

The adherence data is inseparable from the utilization of the self-monitoring form. Completed workouts were determined by calculating the percentage of self-monitoring forms that were returned, so on 81% of days, participants returned the survey.

We do understand the intuitive sense that daily messages are too much of a burden, especially for older adults. However, our data does not seem to suggest that this is the case – at least not to the point of refusing to complete the daily forms.

3. Inclusion of the REDCap survey values, RPEs, and the common exercise modifications the subjects used would be valuable data for inclusion.

Response: *Thank you for your comment. While we agree that Inclusion of the REDCap survey values, RPEs, and the common exercise modifications the subjects are valuable data, the purpose of this manuscript is to report* the effects of a 12-week brief, home-based functional RT program, FAST (*Functional Activity Strength Training)*-2, on functional impairment (the primary outcome) and adherence in older, inactive adults ≥ 65 years of age, with pre-existing walking difficulty.

4. PG 13: Either reverse the order of the OLST and the CST in figure2 and table 2 or reverse it in the text so that the order parallels each other.

Response: *Thank you for your comment. We have incorporated the suggested change and reversed the order of OLST and CST in the text to mirror the order in figure 2 and table 2 (Lines 347-367)*

5. Include discussion on where the subjects land in relation to norms for the functional tests and how this might affect how much they were able to improve, and generally what the scores for the people at baseline and in the control group translate to.

Response: *Thank you for this suggestion. For these tests, there are not well established "normative" values. However, it safe to assume that these participants are on the low side of average, given their pre-existing walking difficulty.*

Instead, what we have included in the text are minimum clinically important differences – i.e., thresholds for change that indicate a clinically important improvement. This section has been updated in text as below: "This is clinically significant as the reported Minimum Clinically Important Difference (MCID) for the 30 second chair stand test is ≥ 2 repetitions for predicting improvement in the 6-minute walk test.(37) and the MCID for FTSTS is 2.3 seconds (39)." (Line 397)
