# Supplementary material for: Brief daily functional strength training to improve functional performance in older adults with mobility disability: A randomized trial
Source: PLoS One. 2026 Mar 12;21(3):e0336748. doi: 10.1371/journal.pone.0336748 (PMC12981469; doi:10.1371/journal.pone.0336748)
Supplement: Attachment — Submitted filename: Response_to_reviewers_auresp_2.pdf [file pone.0336748.s005.pdf]

January 28, 2025,

Dear Editor,

We want to thank you and the reviewers for taking the time to review our revised manuscript titled "Brief daily functional strength training to improve functional performance in older adults with mobility disability: A randomized trial" where we provide experimental evidence that a 12-week (4-minute) , home-based functional resistance training program augmented with goal setting, FAST (*Functional Activity Strength Training)*-2, can enhance adherence and improve functional impairment in older, inactive adults ≥ 65 years of age, with pre-existing walking difficulty. We greatly appreciate your thoughtful review of our response to the points raised by the reviewers. We are pleased to hear that we adequately addressed the comments raised by reviewer # 1 and are hopeful that this second revision addresses the comments mentioned by reviewer # 2 in the prior revision. The new revisions are mentioned in red in the body of the manuscript.

Please find our detailed responses to recent comments by reviewer # 2 below.

1. L131-133: The answer to reviewer 1 comment was inadequately incorporated. Add the additional context that's included in the response to reviewers.

Response: *We thank the reviewer for this thoughtful suggestion and agree that our explanation of the rationale for the 12-week duration of the trial could be improved. We have added additional context as recommended by the reviewer. This section has been updated, and the revised text reads as follows: "This duration was selected as 12 weeks is the typical length of exercise trials. Data suggests that 12 weeks is long enough to allow for physiological changes to occur, both the neural and muscular changes associated with strength training. Initial trials (i.e., those testing a new intervention) typically last 12 weeks to balance participant burden with the ability to test the efficacy of the program. Then, longer trials can be conducted if the 12-week intervention suggested that further investigation was warranted. (Lines 132-137)*

2. L194-197: Add that feet remained flat on floor to prevent rolling of band/ injury.

Response: *Thank you for your comment. We have added the sentence as suggested by the reviewer. This section has been updated in text as follows: "The feet remained flat on the floor to prevent the band from rolling out from under the feet and to prevent injury." (Lines 202-203)*

3. Section "Self-monitoring, feedback, and messaging": Include text from response to reviewers that highlight this is not a burden.

Response: *We have included text from the response to reviewers in the section "Self-monitoring, feedback, and messaging" that highlights it was not a burden. The text has been modified and reads as follows: "The self-monitoring form also included an audio file (with timer) to instruct participants through the exercises. In that way it was less of an "extra" thing to do and more of an integral part of the intervention. The survey was brief, lasting less than 5 minutes each day. Emails also included links to videos of each exercise." (Lines 245-248)*

4. Reviewer 2, #3: The purpose of this manuscript is clear, Inclusion of the requested data either as a table or in text aids this and gives more information about the strain on participants, the movement capacity and ability of the participants, and therefore the feasibility to support adoption of this program. This detail is needed to change current practice.

Response: *We thank the reviewer for this comment. While the purpose of this manuscript is to report* the *effects of a 12-week brief, home-based functional RT program, FAST (Functional Activity Strength Training)-2, on functional impairment (the primary outcome) and adherence in older, inactive adults ≥ 65 years of age, with pre-existing walking difficulty*, *we agree that Inclusion of the common exercise modifications used by the participants and information about REDCap survey values on the reps and RPEs (rate of perceived exertion) is helpful information supporting the feasibility of adoption of the program. We have included information on the RPE scale used in the Materials and Methods section which reads as follows: "The perception of effort was recorded using the 10-point Category-Ratio scale (CR-10)(30). Participants were only asked to report the rate of perceived exertion (RPE) for the chair stands to minimize participant burden. CR-10 scores less than 5 are considered moderate-intensity, with approximate changes in heart rate of 40–60 beats-per-minute. (31)" (Lines 241-245).*

We have now also included information about the modifications used in the exercise sessions, information on the REDCap survey values on repetitions and the RPEs. The included text now reads "Modifications were used in 40.6% of exercise sessions. The reported frequencies of modifications (among those 40% of sessions) for each exercise were as follows: push-ups 70.3%, chair stands 24.1%, rows 12.0%, stair stepping 22.9%. Among the modifications provided, the most common modifications used were: wall pushups, using two hands on knees for the chair stands, using lighter resistance bands for the two arm seated band rows, reducing height of the stepper and using a chair to assist with the stair stepping. Over 12 weeks, the average repetitions increased from 8.1 to 17.5 for pushups, from 7.6 to 16.7 for chair stands, from 10.6 to 27.5 for rows and from 8.7 to 15.8 for stair stepping. While the RPE scores for the chair stands increased slightly from 3.2 to 4.6, it was < 5.0 and within moderate intensity scoring on the CR-10 scale." (Lines 381-390)

5. Reviewer 2, #5: I was able to find values and indicators for the FTSTS, the OLST, 30s chair stand test. Not knowing how these participants compare to the population they represent or a related population is a major limitation.

Response: *We thank the reviewer for this insightful suggestion. We acknowledge that not knowing how the performance times for the FTSTS, OLT and 30 second chair stand test of the participants compared to the related population is a limitation. We have modified the text to include an entire paragraph with information about relative normative values. Additionally, as these "normative" values as not well established for older adults with pre-existing walking difficulty, we have included minimum clinically important differences (MCIDs)– i.e., thresholds for change that indicate a clinically important improvement. We are hopeful that these additions in the "Discussion" section meet the reviewer's expectations. The text now reads as below:*

"Age match normative values for the FTSTS in older adults are well described for community dwelling older adults (with no major walking difficulty). (42) These community norms underestimate expected times in the mobility impaired population of older adults with pre-existing walking difficulty. (43, 44) For older adults with pre-existing walking difficulty, FTSTS time around 15-20 seconds is common, but values ≥16 seconds are often interpreted as indicating elevated fall risk and need for strengthening/balance interventions.(43, 44) In our study, at baseline, the mean FTSTS time for the intervention group was 12.2 (4.6) seconds, and for the control group it was 14.4 (5.3) seconds (p = 0.08). After 12 weeks, the intervention group decreased their FTSTS time by 2.28 seconds more than the control group (95% CI: 0.47-4.09; p = 0.01). Similar to FTSTS, normative values for the 30 second chair stand test are well described for generally healthy, community-dwelling older adults (33)(45), but there are no widely accepted separate norms specifically for those with pre-existing walking difficulty and they likely

overestimate expected performance for this group. Scores below 10-12 often signal increased fall risk and poorer mobility and older adults with pre-existing gait problems have been reported to have mean scores around 8–11 stands.(33)(46) In our study, at baseline, the mean number of chair stands in the intervention group was 9.5 (3.7) and in the control group was 10 (4.3) (p = 0.53). Over 12 weeks, the intervention group increased the number of chair stands by 4.22 repetitions (95% CI: 2.78-5.66) more than the control group (p < 0.001). For older adults with pre-existing walking difficulty, a OLST time of less than 5 to 10 seconds generally indicates a significant balance issue and an increased risk for falls. (47, 48) In our study, at baseline, the mean OLST time in the intervention was 7.3 (7.2) seconds and in the control group it was 7.7 (8.8) seconds (p = 0.82). Over 12 weeks, the intervention group increased their OLST time by 3.57 seconds (95% CI: 0.61-6.53, p = 0.02) compared with the control group. These results suggest significant gains in functional strength, and mobility in this mobility impaired population." (Lines 419-442)
